# Engineering α-carboxysomes into plant chloroplasts to support autotrophic photosynthesis

**DOI:** 10.1038/s41467-023-37490-0

**Published:** 2023-04-25

**Authors:** Taiyu Chen, Marta Hojka, Philip Davey, Yaqi Sun, Gregory F. Dykes, Fei Zhou, Tracy Lawson, Peter J. Nixon, Yongjun Lin, Lu-Ning Liu

**Affiliations:** 1 Institute of Systems, Molecular and Integrative Biology, University of Liverpool, Liverpool, L69 7ZB UK; 2 National Key Laboratory of Crop Genetic Improvement and National Center of Plant Gene Research, Huazhong Agricultural University, 430070 Wuhan, China; 3 Department of Life Sciences, Sir Ernst Chain Building-Wolfson Laboratories, Imperial College London, South Kensington Campus, London, SW7 2AZ UK; 4 School of Life Sciences, University of Essex, Colchester, CO4 4SQ UK; 5 College of Marine Life Sciences, and Frontiers Science Center for Deep Ocean Multispheres and Earth System, Ocean University of China, 266003 Qingdao, China

**Keywords:** Molecular engineering in plants, Rubisco, Synthetic biology, Agricultural genetics

## Abstract

The growth in world population, climate change, and resource scarcity necessitate a sustainable increase in crop productivity. Photosynthesis in major crops is limited by the inefficiency of the key CO_2_-fixing enzyme Rubisco, owing to its low carboxylation rate and poor ability to discriminate between CO_2_ and O_2_. In cyanobacteria and proteobacteria, carboxysomes function as the central CO_2_-fixing organelles that elevate CO_2_ levels around encapsulated Rubisco to enhance carboxylation. There is growing interest in engineering carboxysomes into crop chloroplasts as a potential route for improving photosynthesis and crop yields. Here, we generate morphologically correct carboxysomes in tobacco chloroplasts by transforming nine carboxysome genetic components derived from a proteobacterium. The chloroplast-expressed carboxysomes display a structural and functional integrity comparable to native carboxysomes and support autotrophic growth and photosynthesis of the transplastomic plants at elevated CO_2_. Our study provides proof-of-concept for a route to engineering fully functional CO_2_-fixing modules and entire CO_2_-concentrating mechanisms into chloroplasts to improve crop photosynthesis and productivity.

## Abstract (web summary)

Engineering carboxysomes into crop chloroplasts is a potential route to improve photosynthesis and crop yield. Here, the authors engineer functional CO_2_-fixing modules into tobacco chloroplasts to improve their photosynthesis and productivity.

## Introduction

There is an urgent need to increase global crop productivity in a changing climate to provide sufficient food for the world’s population which is likely to grow to nearly 10 billion by 2050^1,2^. Photosynthesis uses solar energy to convert atmospheric CO_2_ into organic compounds, the essential building blocks for almost all life on Earth. Given its pivotal role in plant growth and development of the global ecosystem, improving photosynthesis has recently emerged as a strategy to underpin step-change improvements in the yields of biomass and major crops^3–5^.

A major rate-limiting step of the photosynthetic process is the carbon fixation reaction catalyzed by the key enzyme ribulose-1,5-bisphosphate carboxylase/oxygenase (Rubisco) in the Calvin–Benson–Bassham cycle^6^. In spite of its biological significance on a global scale and high abundance in the leaf, Rubisco is an inefficient catalyst, due to its slow carboxylation rate and poor ability to discriminate between CO_2_ and O_2_^7–10^. Reaction of Rubisco with the competing O_2_ molecule results in the oxygenation of RuBP that ultimately wastes energy and decreases sugar synthesis during the process of photorespiration^11,12^. To overcome the inherent limitations of Rubisco and reduce “wasteful” photorespiration, many organisms, including C_4_ plants, algae, and many autotrophic bacteria, have evolved CO_2_-concentrating mechanisms (CCM) to internally elevate CO_2_ levels around Rubisco. However, the CCM pathways are absent in the major agricultural C_3_ crops, resulting in a lower photosynthetic efficiency^13^. In this context, engineering a functional CCM into C_3_ crops to enhance CO_2_ fixation and crop yields has recently received increasing attention in the area of food security^14–17^.

Cyanobacteria and many proteobacteria employ carboxysomes as the central CO_2_-fixing organelles of the bacterial CCM^18,19^. The carboxysome encapsulates a Rubisco enzyme with a fast turnover rate and carbonic anhydrases (CA) using a polyhedral protein shell that consists of multiple protein homologs in the form of hexamers, pentamers, and trimers^11,18,19^. According to the type of Rubisco, carboxysomes can be categorized into two lineages: α-carboxysomes that possess Form-1A Rubisco and β-carboxysomes that contain plant-like Form-1B Rubisco^19^. In the carboxysome-based CCM, active HCO_3_^−^ transporters pump external HCO_3_^−^ into the cell, generating an accumulated HCO_3_^−^ pool in the cytosol^20–22^. HCO_3_^−^ can passively diffuse across the semi-permeable shell of the carboxysome^23^, and is then dehydrated to CO_2_ by the carboxysomal CA, permitting a substantial elevation of CO_2_ levels around the catalytic sites of Rubisco to favor carboxylation^24^. Overall, the naturally occurring architecture and permeability of the carboxysome provide the foundation for enhanced carbon assimilation and allow carboxysomes to play an important role in the natural ocean and freshwater carbon cycle^11,25,26^.

**Fig. 1:**
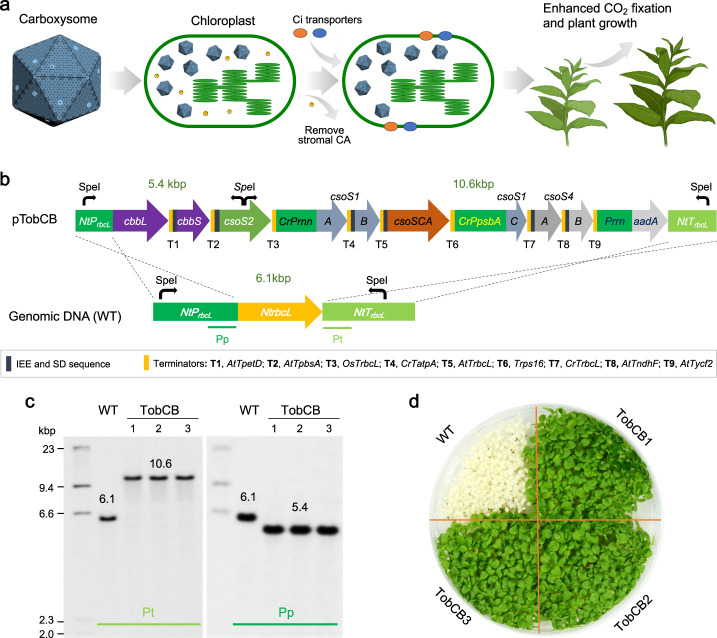
Synthetic engineering of α-carboxysomes into tobacco chloroplasts. **a** Schematic representation of the strategies of introducing carboxysomes and bicarbonate transporters into plant chloroplasts and eliminating chloroplastic CA to install a complete CCM for enhanced photosynthetic carbon fixation and plant yields. **b** Gene organization of α-carboxysome-expressing construct for tobacco chloroplast expression and the *rbcL* locus in the wild-type (WT) tobacco chloroplast genome. The α-carboxysome-expressing construct contains nine genes coding Rubisco (*cbbL* and *cbbS*), the linker protein CsoS2 (*csoS2*), carbonic anhydrase (*csoSCA*), shell hexamers (*CsoS1A/B/C*) and pentameric proteins (*csoS4A/B*). The genes were grouped into three operons driven by *NtPrbcL* (native promoter of *rbcL* in *Nicotiana tabacum*), *CrPrrn* (promoter of ribosomal RNA in *Chlamydomonas reinhardtii*), and *CrPpsbA* (promoter of *psbA* in *C. reinhardtii*), respectively. The Streptomycin/Spectinomycin adenylyltransferase gene (*aadA*) was driven by the tobacco plastid rRNA operon promoter (P*rrn*)^73^. Intercistronic Expression Elements (IEE), SD (Shine-Dalgarno) sequence, and Terminators (T) were listed. *At*, *Os*, and *Cr* indicate *Arabidopsis thaliana*, *Oryza sativa*, and *C. reinhardtii*, respectively. **c**, **d** Southern blot analysis (**c**) and seed germination (**d**) verified the successful transgene integration and homoplasmy of the three transplastomic plants obtained. The genomic DNA was digested by SpeI and hybridized with Digoxygenin-labeled probes of the promoter (Pp) and terminator (Pt) of *rbcL* in *N. tabacum* as indicated in (**b**). Seed germination was performed on Murashige and Skoog (MS) medium containing 500 mg L^–1^ spectinomycin. Source data are provided as a Source Data file.

Given the self-assembly of the carboxysome and its significance in enhancing CO_2_ fixation, introducing functional carboxysomes and eventually the entire CCM into crop chloroplasts has great potential for improving crop photosynthesis (an increase of up to 60% has been estimated with the incorporation of a complete CCM^1,14^) and yields^14–17,27,28^ (Fig. 1). This endeavor has been further spurred on by the success in heterologous engineering of carboxysomes in bacterial hosts^29–32^ and CCM in Rubisco-dependent *Escherichia coli* (*E. coli*) to support autotrophic growth of bacterial cells^33^. Successful attempts have also been made in engineering the chloroplasts of the model crop plant tobacco to express cyanobacterial Form-1B Rubisco (to replace the plant’s endogenous Rubisco)^34,35^, the Rubisco large subunit^36^, or β-carboxysome shell proteins^37^. Recent studies further reported the construction of simplified α-carboxysomes (containing Form-1A Rubisco along with the linker CsoS2 and the shell protein CsoS1A) derived from a cyanobacterium *Cyanobium marinum* PCC 7001 in tobacco chloroplasts^38^. However, an entire functional carboxysome including CA and all the structural components have not yet been engineered into plant chloroplasts.

The α-carboxysome from the proteobacterium *Halothiobacillus neapolitanus* (*H. neapolitanus*) represents one of the best-characterized carboxysomes involved in the global carbon cycle. Recent studies have provided advanced knowledge about its protein composition, self-assembly, shell-cargo encapsulation, three-dimensional structure, and internal Rubisco organization^11,31,39–42^. The extensive information available for *H. neapolitanus* α-carboxysome assembly and functionality makes it an ideal candidate for engineering carbon-assimilating modules in diverse non-native organisms^29–31,39^, for example, with the intent of enhancing H_2_ production^43^ and expressing a more active Rubisco in tobacco chloroplasts^44^.

Here, we transform the full set of α-carboxysome components, encoded by nine genes in the *cso* operon (CarboxySOme) of the *H. neapolitanus* genome, into *Nicotiana tabacum* (*Nt*, tobacco) chloroplasts, and demonstrate that the transplastomic plants can produce intact, fully functional α-carboxysomes that support plant growth and autotrophic photosynthesis. This study represents a major step forward toward installing an entire functional CCM into chloroplasts to enhance crop photosynthesis and yields.

## Results

### Vector construction and chloroplast transformation

The α-carboxysome from *H. neapolitanus* represents a promising target for synthetic engineering in heterologous bacterial hosts^29–31,33^. To examine the feasibility of expressing and generating functional α-carboxysomes in tobacco chloroplasts, the α-carboxysome genes encoding *H*. *neapolitanus* Form-1A Rubisco (*Hn*Rubisco) large and small subunits (*cbbL*, *cbbS*), the linker protein CsoS2, shell pentamers (*csoS4A*/*B*), CA (*csoSCA*), shell hexamers (*csoS1A/B/C*), as well as the necessary elements for gene transcription and translation in chloroplasts including terminators, intercistronic expression elements (IEE), Shine-Dalgarno (SD) sequences, and an *aadA* gene conferring spectinomycin resistance, were clustered into three operons in a chloroplast transformation plasmid pTPTR, to generate the pTobCB vector (Fig. 1b and Supplementary Data 1). A 6x-Histidine tag was fused to the C-terminus of CbbL to facilitate the differentiation of *Nt*RbcL and *Hn*CbbL in transgenic plants. The pTobCB vector was then transformed into tobacco chloroplasts via biolistic bombardment to replace the endogenous tobacco Rubisco large subunit gene (*NtrbcL*) and express the full set of α-carboxysome protein components encoded by nine genes in the *cso* operon (Fig. 1b). Positive transgenic lines were obtained after several rounds of selection and regeneration. These transplastomic plants were then grown autotrophically in soil in air with 1% (v/v) CO_2_ supplementation to flowering and seed collection, and three distinct transplastomic lines were reserved for further analysis.

To verify the homoplasmy of the transplastomic plants, we first selected SpeI to digest the whole carboxysome-expression cassettes (over 12 kbp) in the genomic DNA, resulting in two fragments of 5.4 kbp and 10.6 kbp, respectively (Fig. 1b). Southern blotting analysis using DNA fragments specific for the 5’ UTR and 3’ UTR of *NtrbcL* as probes revealed that the sizes of the target fragments were shifted from 6.1 kbp (wild type, WT) to 5.4 kbp and 10.6 kbp, respectively, demonstrating complete replacement of the WT fragments in the transgenic lines (Fig. 1c). Moreover, seed testing analysis showed that all the transgenic seeds were green, whereas the WT seeds were completely white on plates containing 500 mg L^−1^ spectinomycin (Fig. 1d). Collectively, our results confirmed the full integration of the carboxysome operons into the WT tobacco chloroplast genome, resulting in homoplasmic chloroplast transformants.

### Expression of α-carboxysome proteins in transgenic plants

**Fig. 2:**
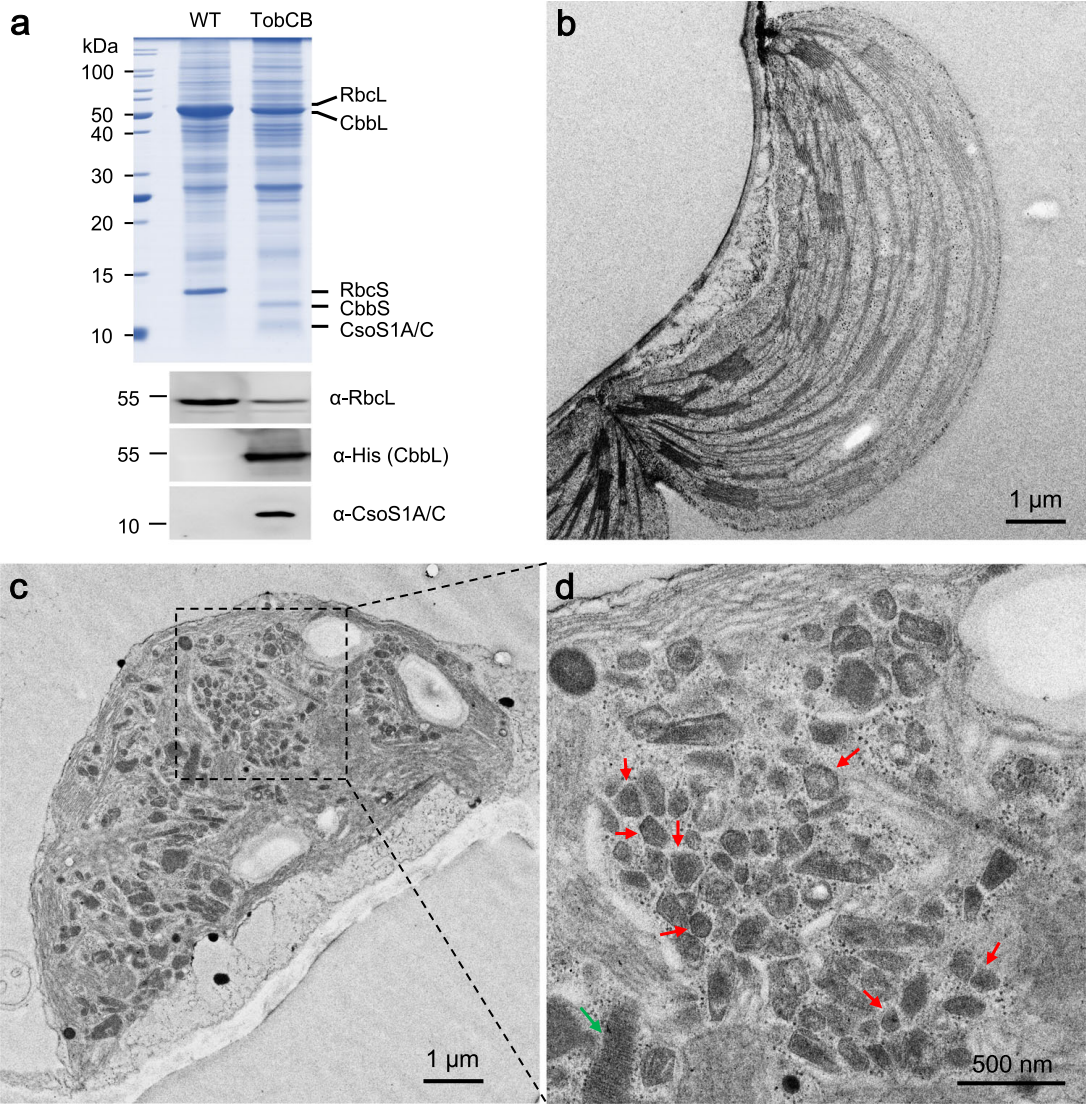
Expression of α-carboxysome structures in tobacco chloroplasts. **a** SDS-PAGE (top) and immunoblot analysis (bottom) of total soluble proteins of the WT and TobCB leaves indicated the expression of α-carboxysome proteins in the chloroplast transformant TobCB. **b** Representative transmission electron micrograph (EM) of leaf sections from WT tobacco. **c**, **d** EM of leaf sections from the chloroplast transformant TobCB. Carboxysome-like structures were clearly observed in the chloroplasts of the TobCB transgenic line, whereas the WT chloroplasts have normal architectures (**b**). **d** higher magnification view of the carboxysome-like structures in TobCB chloroplasts indicated in (**c**). Canonic polyhedral carboxysome structures were indicated in red arrows. Rubisco assemblies are indicated in green arrow. See also Supplementary Fig 2. At least three biologically independent samples were analyzed. Source data are provided as a Source Data file.

Sodium dodecyl sulfate-polyacrylamide gel electrophoresis (SDS-PAGE) and immunoblot analysis of the transplastomic tobacco (TobCB) leaf extracts (with equal loading) revealed the successful expression of *Hn*CbbL, *Hn*CbbS, and the major shell proteins CsoS1A/C in transplastomic leaves (Fig. 2a). Along with *Hn*CbbS expression, the content of endogenous *Nt*RbcS (~15 kDa) was greatly reduced in the transformants, suggesting that free *Nt*RbcS may be largely degraded in the absence of *Nt*RbcL in the tobacco chloroplast^38,45–47^. As the RbcL antibody used in this study was unable to differentiate *Nt*RbcL and *Hn*CbbL, immunoblot analysis using an anti-6x-Histidine tag antibody confirmed the expression of *Hn*CbbL in chloroplast transformants (Fig. 2a).

SDS-PAGE of TobCB leaf extracts also indicated that the Rubisco content was less in transgenic chloroplasts than in the WT (Fig. 2a). Quantification of the Rubisco content, by examining the regression of Rubisco activity against the concentration of the Rubisco inhibitor carboxyarabinitol-1,5-bisphosphate (CABP)^48^, further revealed that the Rubisco content in the transgenic chloroplasts was ~30% the *Nt*Rubisco content in WT tobacco chloroplasts (Table 1 and Supplementary Fig. 1) and slightly less than that in the *Hn*Rubisco-expressing tobacco chloroplasts;^44^ however, this value is greater than the Rubisco content in transgenic tobacco chloroplasts that produce simplified cyanobacterial α-carboxysomes (~10% the *Nt*Rubisco level in WT chloroplasts)^38^.

**Table 1:** Catalytic parameters of purified carboxysomes from engineered chloroplasts *H. neapolitanus*, and *E. coli*

Parameters	CB^Tob^	CB^Halo^	CB^Ecoli^
*k*_*cat*_^*C*^ (μmol mg^–1^ min^–1^)	3.7 ± 0.1	3.6 ± 0.1	4.2 ± 0.2
*K*_*C*_ (μM)	182.0 ± 14.5	207.5 ± 19.4	206.1 ± 21.7

Data are presented as mean ± SD (*n* = 3 independently biological replicates).

### Formation of α-carboxysome structures in transgenic chloroplasts

To evaluate whether the expressed protein components could form α-carboxysomes in the chloroplasts of transgenic plants, we performed thin-section transmission electron microscopy (TEM) of tobacco leaves. Compared to the typical chloroplast ultrastructure of WT tobacco (Fig. 2b), the TobCB chloroplasts contained numerous electron-dense, higher-ordered particles spaced between the thylakoid grana stacks (Fig. 2c, d and Supplementary Fig. 2). Many of these particles exhibited a polyhedral structure with straight edges and vertices, resembling the α-carboxysome structures in native or heterologous bacterial hosts^31,39,49,50^.

Closer inspection revealed that the α-carboxysome-like features had variable architectures (Supplementary Fig. 2). The large protein aggregates are reminiscent of the Rubisco assemblies formed in the presence of scaffolding proteins (CcmM for β-carboxysomal Rubisco)^35,36^ or the procarboxysomes in β-carboxysome biogenesis^51^. The elongated structures have also been observed in transgenic chloroplasts that produce simplified carboxysomes in the absence of the shell vertex proteins CsoS4A/B^38^, in a *H*. *neapolitanus* mutant that was depleted of CsoS4A/B^52^, as well as in cyanobacterial mutants that lack carboxysome shell vertex proteins^51,53,54^.

### Characterization of tobacco chloroplast-produced α-carboxysomes

To further determine the structural and catalytic properties of the chloroplast-produced α-carboxysomes (CB^Tob^), we purified the α-carboxysomes from transgenic leaves using 10-50% (w/w) sucrose density-gradient centrifugation^38^ (Supplementary Fig. 3). SDS-PAGE, immunoblot, TEM, and Rubisco activity assays indicated that intact, functional α-carboxysomes were enriched at the interfaces of the 30%, 40%, and 50% sucrose fractions, consistent with native α-carboxysomes from *H. neapolitanus*^39^. The α-carboxysomes at the 50% sucrose fraction exhibited the highest abundance and activity and were thus chosen for the following characterization (Supplementary Fig. 3).

**Fig. 3:**
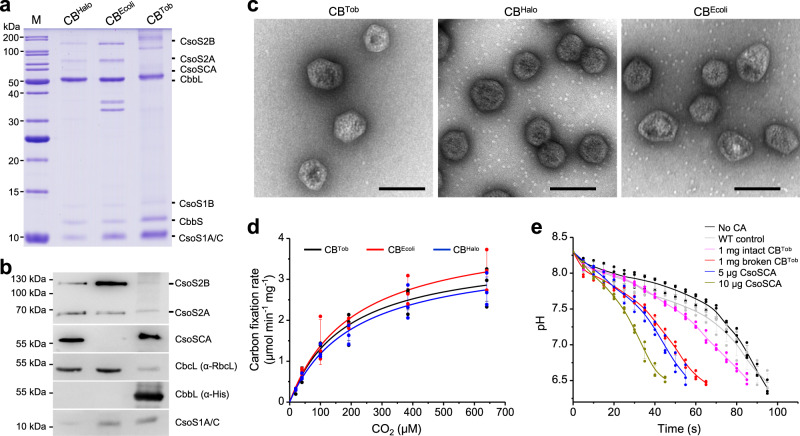
Determination of the assembly and enzymatic activity of the purified carboxysomes. **a**, **b** Representative SDS-PAGE (**a**) and immunoblot analysis (**b**) of native α-carboxysomes purified from *H. neapolitanus* (CB^Halo^), synthetic α-carboxysomes from *E. coli* (CB^Ecoli^)^31^, and chloroplast-expressed α-carboxysomes from tobacco (CB^Tob^). At least three biologically independent samples were analyzed. All the protein amounts in SDS-PAGE and immunoblot analysis are 20 µg except for the immunoblot analysis of CsoS2, in which the total protein amounts of CB^Halo^, CB^Ecoli^, and CB^Tob^ are 2 µg, 2 µg, and 20 µg. **c** EM images of isolated carboxysomes. The CB^Tob^ are structurally variable, with an average diameter is 154.1 ± 36.9 nm (*n* = 204), which is slightly greater than those of CB^Halo^ (~130 nm)^39^ and CB^Ecoli^ (120–140 nm)^29,31^. Scale bar: 200 nm. **d** Carbon fixation assays of CB^Tob^ under different CO_2_, in comparison with CB^Halo^ and CB^Ecoli^. *k*_cat_^c^ of CB^Tob^, CB^Halo^, and CB^Ecoli^ were 3.7 ± 0.1 μmol mg^–1^ min^–1^, 3.6 ± 0.1 μmol mg^–1^ min^–1^ and 4.2 ± 0.2 μmol mg^–1^ min^–1^ (*n* = 3), respectively. *K*_*C*_ of CB^Tob^, CB^Halo^, and CB^Ecoli^ were 182.0 ± 14.5 μM, 207.5 ± 19.4 μM, and 206.1 ± 21.7 μM (*n* = 3). Data were normalized against the content of Rubisco. See also Table 1. **e** CA activity assays to measure the pH changes of the assay buffer as a function of time due to the conversion of CO_2_ to HCO_3_^–^ catalyzed by CA. Adding purified CB^Tob^ (ruptured CB^Tob^, red; intact CB^Tob^, magenta) into the assay buffers caused a faster decrease in the assay buffer pH than no-CA control (black), WT control (gray, tobacco leaf extract obtained following the carboxysome purification procedure), and ruptured CB^Tob^ resulted in a faster reduction in the buffer pH compared to intact CB^Tob^, indicating that the encapsulated CA within CB^Tob^ were catalytically active. Compared with the CA activities of purified CsoSCA (5 μg, blue; 10 μg, dark green), the CA activity of 1 mg purified CB^Tob^ equivalents to that of 5 μg purified CsoSCA (blue). See Supplementary Fig. 7 for purified CsoSCA. Data are presented as mean ± SD from three independent biological replicates. Source data are provided as Source Data file.

SDS-PAGE and immunoblot analysis revealed the presence of CbbL, CbbS, CsoS1A/B/C, and CsoSCA in CB^Tob^ (Fig. 3a, b). CsoS2A and CsoS2B were not visible by SDS-PAGE, but were detectable by immunoblot analysis using an anti-CsoS2 antibody. The immunoblot signal for CsoS2A and CsoS2B of CB^Tob^ was weaker than that of native α-carboxysomes purified from *H. neapolitanus* (CB^Halo^) and synthetic α-carboxysomes from *E. coli* (CB^Ecoli^), indicating the reduced content of both isoforms of CsoS2 in CB^Tob^. Mass spectrometry analysis on the purified CB^Tob^ identified all nine α-carboxysome components, including Rubisco (CbbL, CbbS), CA (CsoSCA), the linker protein CsoS2, shell hexamers CsoS1A/B/C, and shell pentamers CsoS4A/B (Supplementary Data 2). We further performed relative quantification analysis of the protein abundance of individual components in CB^Tob^ using label-free mass spectrometry in comparison with those in CB^Halo^ and CB^Ecoli^ (Supplementary Table 1)^39^. Based on SDS-PAGE profiles and label-free mass spectrometry results (normalized to the abundance of the shell proteins CsoS1A/C), CB^Tob^ has a reduced content of Rubisco, CA, and CsoS2 compared with CB^Halo^, which is consistent with SDS-PAGE and the immunoblot analysis (Fig. 3a, b and Supplementary Fig. 4).

Negative-staining TEM of purified CB^Tob^ showed the canonical α-carboxysome structures consisting of the polyhedral outer shell and encapsulated cargos. The diameter of CB^Tob^ is 154.1 ± 36.9 nm (*n* = 204) (Fig. 3c and Supplementary Fig. 5), slightly greater than those of CB^Halo^ (~130 nm)^39^ and CB^Ecoli^ (120–140 nm)^29,31^ as well as simplified α-carboxysomes produced previously in tobacco (~100 nm)^38^. In addition, the purified CB^Tob^ showed structural heterogeneity in shape and size (Supplementary Fig. 5), in agreement with the leaf ultra-thin-section results (Supplementary Fig. 2) and chloroplast-produced simplified α-carboxysomes^38^. Whether the incorporation of other carboxysome-associated components like CsoS1D will improve the structural rigidity of the chloroplast-expressed α-carboxysomes remains to be tackled.

Rubisco ^14^CO_2_-fixing assays were conducted as a function of CO_2_ concentration to evaluate the carboxylation activity of purified CB^Tob^. The results indicated that Rubisco within the purified CB^Tob^ from tobacco chloroplast transformants has a comparable catalytic rate constant for carboxylation (*k*_cat_^c^) and affinity for CO_2_ (*K*_*C*_) to those of purified CB^Halo^ and CB^Ecoli^^31,39^ (Fig. 3d and Table 1). Assays using different pH buffers showed that the CO_2_-fixing activities of CB^Tob^ and free Rubisco rose slightly with the increase in buffer pH below pH 7.7 and 7.4, respectively, and dramatically decreased upon increasing the pH value to pH 9.2 (Supplementary Fig. 6). Compared with free Rubisco, CB^Tob^ exhibited a retarded decrease in CO_2_-fixing activities with the increase in pH (the shift of pH peak from 7.4 to 7.7). It suggests that the protein shell could generate a more acidic pH microenvironment within CB^Tob^ than the external pH, in good agreement with previous observations^55^, implicating the functional integrity of the CB^Tob^ shell to provide a distinct interior pH environment (Supplementary Fig. 6). Meanwhile, free Rubisco showed higher activity than when present in CB^Tob^ (Supplementary Fig. 6a), which is similar to previous data^56^. Moreover, we determined the CA activities of CB^Tob^ for CO_2_ hydration in vitro using a potentiometric method^57–60^ to monitor the pH changes of the assay buffer due to the conversion of CO_2_ to HCO_3_^-^ catalyzed by CA. The results showed that the assay buffer pH displayed a faster decrease with the addition of CB^Tob^ (intact and ruptured CB^Tob^) than that of the controls lacking CA (no addition of CA or WT tobacco leaf extract obtained following the carboxysome purification procedure), indicating that the encapsulated CA within CB^Tob^ was catalytically active (Fig. 3e). Consistent with previous finding^61^, the ruptured CB^Tob^ samples with CA being exposed to external buffer resulted in a faster reduction in the buffer pH compared to intact CB^Tob^ in which CA are still mostly encapsulated by shells, implicating the functional intactness of the protein shell of CB^Tob^ in providing a barrier for CO_2_ passage. Collectively, our results demonstrated the generation in tobacco chloroplasts of α-carboxysomes with at least nine groups of building components as well as catalytically active Rubisco and CA.

### Chloroplast-generated α-carboxysomes support plant growth at elevated CO_2_

**Fig. 4:**
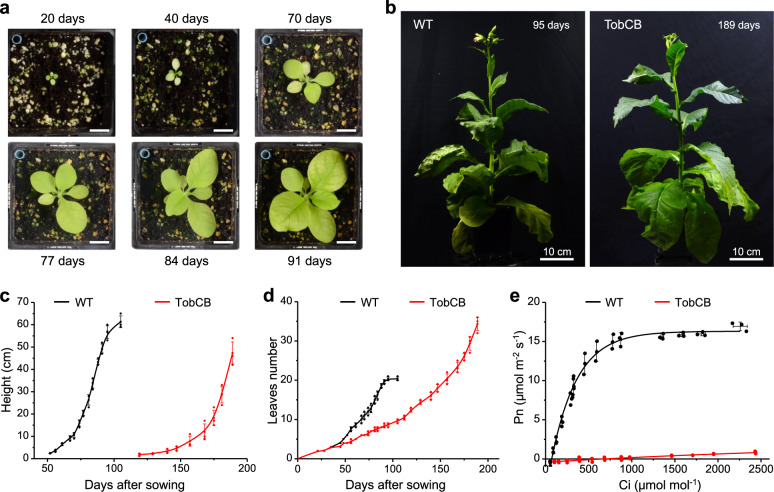
Carboxysome-mediated plant growth. **a** The growth of TobCB in air supplemented with 1% CO_2_ after sowing. Scale bar: 2 cm. **b** Growth phenotypes of WT (95 days after sowing) and transgenic plants (189 days after sowing) in air supplemented with 1% CO_2_. **c**, **d** Plant growth curves indicated as height (**c**) and leaves number (**d**) after sowing. **e** Leaf gas-exchange analysis of net CO_2_ photosynthesis rate (Pn) of WT and TobCB as a function of intercellular CO_2_ pressures (Ci) at 25 °C and 1200 μmol photons m^−2^ s^−1^ light density, measured by gas exchange of attached leaves. The measurements of WT and TobCB were conducted at 65 and 120 days after sowing, on which the plants of WT and TobCB showed a similar height and a similar size of leaves. Data are presented as mean ± SD (*n* = 3 independent biological replicates). Source data are provided as a Source Data file.

The TobCB transgenic lines were planted and grown in soil to examine their growth phenotypes. Seeds of the TobCB plants could be germinated, and the TobCB plants demonstrated autotrophic growth and a full life cycle in air supplemented with 1% CO_2_ (v/v) (Fig. 4a, b), but were unable to grow in ambient air (~400 ppm CO_2_). At 1% CO_2_, the transgenic plants showed a slower growth rate than WT plants (Fig. 4c, d) and the *Hn*Rubisco-expressing tobacco plants^44^. We note that although the production of CB^Tob^ did not cause marked effects on the chloroplast ultrastructure (Fig. 2), the Rubisco and chlorophyll contents were lower in transgenic lines than in WT plants, which might be attributed to some extent to the slower growth rate compared with WT plants (Fig. 4 and Table 2). Gas-exchange analysis indicated the compensation point of the transgenic lines was nearly 600 ppm (Fig. 4e), significantly higher than that of WT plants but very similar to the tobacco lines producing simplified α-carboxysomes^38^. Despite the TobCB plants showing a higher Rubisco content (~30% of WT) and expressing nine carboxysome components including shell vertex proteins and carboxysomal CA to ensure permeability and catalytic activity, the net CO_2_ assimilation rate of the transgenic lines was still very low at 2500 ppm CO_2_ and insufficient to support autotrophic growth in ambient air (Fig. 4e). These results may suggest that the HCO_3_^−^ concentration within the chloroplast could be an essential factor for efficient carbon fixation in carboxysome-expressing plants, and that the endogenous chloroplastic CA might lead to CO_2_ leakage resulting in an insufficient HCO_3_^−^ supply for carboxysomes^60^. Nevertheless, the growth of transgenic plants at 1% CO_2_ indicated that the catalytic activities of the heterologously engineered α-carboxysomes in tobacco chloroplasts could support plant photosynthesis.

**Table 2:** Comparison of the Rubisco content, protein content, and chlorophyll content of WT and TobCB leaves

Parameters		WT	TobCB
Rubisco content (μmol m^−2^)		6.6 ± 0.3	1.9 ± 0.0**
Soluble protein content (g m^−2^)		1.8 ± 0.2	1.4 ± 0.3
Chlorophyll content	Chl *a* (mg m^−2^)	146.7 ± 7.2	109.4 ± 13.9**
	Chl *b* (mg m^−2^)	48.8 ± 0.3	34.9 ± 2.7**
	Total Chl (mg m^−2^)	195.5 ± 6.9	144.4 ± 16.6**

*P* = 7.3716e^−06^ for Rubisco content, *P* = 0.0608 for soluble protein content, *P* = 0.0072 for Chl a, *P* = 0.0004 for Chl b and *P* = 0.0039 for total Chl. **P < 0.01 (one-sided *t* test). Data are presented as mean ± SD (*n* = 3 independently biological replicates). Source data are provided as Source Data file.

## Discussion

How to enhance photosynthetic efficiency in a sustainable manner is a central question to improve crop productivity and so address the global food security challenge. Here, we have transformed a full set of genes encoding α-carboxysome proteins from a chemoautotrophic bacterium *H. neapolitanus* into tobacco chloroplasts to replace the endogenous tobacco *rbcL* gene in the chloroplast genome. We have demonstrated that all the α-carboxysome building and enzymatic components, including the core enzymes Rubisco and CA, the linker protein CsoS2, shell hexamers CsoS1A/B/C, as well as shell pentamers CsoS4A/B, can be expressed and self-assembled in chloroplasts without any cognate chaperones, resulting in the formation of intact, functional α-carboxysomes. We also evaluated the structural and functional similarity between the chloroplast-produced α-carboxysomes, natural α-carboxysomes from the original host and synthetic α-carboxysomes produced in *E. coli* and showed that the chloroplast-produced α-carboxysomes supported the autotrophic growth of transgenic lines in air supplemented with 1% CO_2_. Our study provides important evidence for the self-assembly nature of carboxysome proteins in eukaryotic organisms and the feasibility of engineering intact, functional carboxysomes without any cognate assembly and activation factors, which is an important milestone in producing a functional CCM in C_3_ plant chloroplasts to improve plant growth.

Recent work has demonstrated the production of simplified α-carboxysomes derived from *Cyanobium marinum* PCC 7001 in tobacco chloroplasts^38^. In contrast to the simplified α-carboxysomes that contain only Rubisco, CsoS2, and CsoS1A, CB^Tob^ generated in this work also includes additional cargo and structural components that are important for α-carboxysome assembly and function. In particular, CA is crucial for providing elevated CO_2_ levels near the catalytic sites of Rubisco to facilitate carboxylation within the carboxysome^56^. The presence of CsoS4A and CsoS4B shell vertex proteins promotes the formation of more regular polyhedral carboxysomes and might function as a CO_2_ leakage barrier in the α-carboxysome shell^51–53^, although some elongated carboxysome structures were discerned in the chloroplast transformants (Fig. 2c, d and Supplementary Figs. 2 and 4). In addition, our transplastomic leaves had approximately threefold the Rubisco content of the simplified α-carboxysome-expressing transgenic leaves (Table 2). These advances provide stepwise improvements on the construct design to generate complete carboxysome structures with enhanced catalytic performance.

The TobCB transplastomic lines were incapable of growth in ambient air, necessitating further optimization of the construct design and chloroplast engineering to improve protein expression, modulate carboxysome assembly and structure, maintain correct chloroplast function, as well as the installation of active bicarbonate transporters and elimination of endogenous chloroplastic CA. First, protein stoichiometry is essential for bacterial microcompartment assembly and functionality^39,62–64^. Although all the α-carboxysome proteins have been expressed in tobacco chloroplasts, their expression levels and stoichiometry in CB^Tob^ remain to be evaluated and fine-tuned to ensure proper assembly of α-carboxysomes in the chloroplast environment. Second, the native *H. neapolitanus* α-carboxysome possesses two isoforms of CsoS2, translated via programmed ribosomal frameshifting^39,65^. CsoS2A is a truncated form that lacks the C-terminal region responsible for carboxysomal shell anchoring, compared to the full-length CsoS2B. Our results showed that both CsoS2A and CsoS2B were expressed in tobacco chloroplasts and assembled within the formed carboxysomes, suggesting that the programmed ribosomal frameshifting elements derived from bacteria could be recognized in the plant chloroplast transcription and translation systems (Fig. 3b). However, the amount of CsoS2 was significantly decreased in CB^Tob^ (Fig. 3a, b and Supplementary Fig. 4), which may lead to the reduced Rubisco encapsulation and overall carboxysome protein stoichiometry^42^. Codon or vector optimization may be required to modulate the protein stoichiometry and minimize the structural heterogeneity to obtain a more defined and functional carboxysome. Another factor that should be considered is the number of IEE sequences to be integrated into the carboxysome gene cassette, as excessive expression of the IEE in the chloroplast may cause negative effects on endogenous mRNA stabilization and chloroplast function^66^. Likewise, the addition of the shell proteins CsoS1D and Rubisco activases (CbbQ/O) may alter carboxysome permeability, rigidity, and function^29,31,67,68^.

Engineering fully functional carboxysomes in the chloroplast is an important first step to engineer a CCM in chloroplasts to improve crop photosynthesis^13,14^. Installing active CCM pathways requires the introduction of bicarbonate transporters in the chloroplast inner membrane and the elimination of endogenous chloroplastic CA activities to efficiently accumulate HCO_3_^−^ in the chloroplast stroma for carboxysome function. It has been predicted that the HCO_3_^–^ concentration will reach 2–3 mM after removing CA from the chloroplast stroma and will further increase to 5 mM with the incorporation of HCO_3_^–^ transporters, such as BicA and SbtA/B^13,38,69^. Recent advances in the structure, function and engineering of BicA and SbtA/B HCO_3_^–^ transporters^20,21,70,71^ as well as eliminating chloroplast stromal CA activity in tobacco by CRISPR/Cas9 mutagenesis^60^ or using an antisense RNA approach^72^ have paved the way towards optimizing the CO_2_-assimilation capacity and growth of carboxysome-engineering plants. The transgenic lines generated in this study will facilitate further development of carboxysome engineering and provide an ideal host for installing fully functional CCM into chloroplasts to improve crop plant photosynthesis and productivity.

## Methods

### Vector construction, chloroplast transformation, and southern blotting

The whole expression cassette including the *cbbLS*, *csoS2*, *CsoSCA*, *csoS4AB*, and *csoS1ABC* genes was clustered into three different operons in a chloroplast transformation plasmid (pTPTR, a plasmid of Tobacco Plastid Transformation of RbcL), as described in Fig. 1b. As the α-RbcL antibody used in this study was not able to differentiate between *Nt*RbcL and *Hn*CbbL, a coding sequence of 6x-His tag was fused to the 3’ end of *HnCbbL* to distinguishing between *Nt*RbcL and *Hn*CbbL. One terminator was designed after each gene to stabilize mRNA, and an intercistronic expression element (IEE) and a Shine-Dalgarno sequence (SD) was added to the front of downstream gene in each operon to increase the translational efficiency^73,74^. Three operons were synthezied (GenScript, Nanjing, China, Supplementary Data 1) and were assembled to pEASY-Blunt Zero (TransGen Biotech, Beijing, China) with up/down homologous fragments of endogenous *rbcL* and antibiotic-resistant gene (*aadA*) by Gibson Assembly method (E5510S, NEB, www.neb.uk.com) to generate the final construct (pTobCB) to replace endogenous *rbcL* by homologous recombination. The primers used in this study are listed in Supplementary Table 2.

A total of 20 μg of plasmid DNA was coated with 3.5 mg gold particle (0.5 mg per shot) according to the standard protocol^44,73^. Wild-type (WT) tobacco (*Nicotiana tabacum cv*. Petit Havana) was cultured on Murashige and Skoog (MS) agar with 30 g L^−1^ sucrose^75^, and 40-day old leaves were chosen for biolistic bombardment according to the manual (PDS-1000/He Particle Delivery System, Bio-Rad, https://www.bio-rad.com). The bombarded leaves were cut into 5 mm × 5 mm size and cultured on RMOP medium (MS with 30 g L^−1^ sucrose, 500 L^−1^ mg spectinomycin, 1 mg L^−1^ 6-benzylaminopurine, 0.1 mg L^−1^ naphthaleneacetic acid, 1 mg L^−1^ thiamine-HCl, 100 mg L^−1^ myo-inositol, 3 g L^−1^ phytagel, pH 5.8) for selection. The positive transforms were cut into small pieces for second-round regeneration on the same medium^76^. The shoots from the second-round regeneration were cut and transferred to a Rooting medium (1/2 basic MS medium containing 500 mg L^−1^ spectinomycin, 30 g L^−1^ sucrose and 0.3% (w/v) phytagel) and then transplanted into soil pots for growth in an incubator (Sanyo, Japan) at ~150 μmol photons m^−2^ s^−1^ (white light) in air supplemented with 1% CO_2_ (v/v). The seeds were sterilized with 75% (v/v) ethanol for 5 min and then cultured on Rooting medium to check the segregation of the chloroplast in transgenic lines.

The genomic DNA of tobacco was extracted following the previously reported method^77^. Specifically, one gram of tobacco leaves were gridded by liquid nitrogen and the leaf powder was immediately mixed with pre-boiled 500 μL extraction buffer (1.5% (w/v) CTAB (cetyltrimethylammonium bromide), 75 mM Tris-HCl, 1.05 M NaCl, 15 mM EDTA pH 8.0) and then were incubated at 65 °C for 10 min. Overall, 500 μL chloroform was added to the leaf homogenate and thoroughly mixed. The sample was centrifuged at 12,000×*g* for 10 min and the supernatant was transferred to a new 1.5-mL tube. In all, 2/3 volume of isopropanol was loaded to the supernatant and then the samples were centrifuged at 12,000×*g* for 10 min to pellet DNA. The resulting DNA was pre-eluted in 200 μL 1 M NaCl and 5 μL RNAse (Invitrogen, 12091021) at 65 °C for 5 h. Overall, 600 μL pre-cooled ethanol was added to the completely eluted sample, followed by centrifugation at 12,000×*g* for 10 min to pellet DNA. The pellet was washed with 500 μL 75% ethanol again and was eluted with 100 μL H_2_O after the ethanol was completely dry. The DNA concentration was quantified using a Nanodrop Ds-11 (DeNovix, USA). About 3 μg genomic DNA was digested by SpeI and then separated by 1% (v/v) agarose gel electrophoresis. After depurination, denaturing, and neutralization, DNA was transferred to Hybond-N^+^Membrane (GE Healthcare Life Sciences, USA) overnight by the Capillary method^78^. The membrane was then fixed at 80 °C for 2 h. The probes were amplified by the PCR method and then labeled by DIG Probe Synthesis Kit according to the manual (Roche, Basel, Switzerland). After pre-hybridization for 5 h, the membrane was hybridized with the DIG probe at 42 °C overnight and the free probe was removed by three times washing with the 1× SSC buffer (saline-sodium citrate, 0.15 M NaCl and 15 mM sodium citrate, and 0.1% (w/v) SDS) at 56 °C for 10 min. The membrane was blocked with block solution (Roche, Basel, Switzerland) and then immuno-blotted with anti-digoxigenin alkaline phosphatase antibody (Roche, Basel, Switzerland). After two rounds of membrane washing and balanced with detection buffer (Roche, Basel, Switzerland), the membrane was finally soaked with 1 mL CDP-Star®, ready-to-use (Sigma-Aldrich, Missouri, USA), and imaged with Image Quant LAS 4000 (GE Healthcare Life Sciences, USA).

### Plant growth and gas exchange

The transgenic plants were transferred to soil and grown in the greenhouse until the seeds could be harvested. For growth tracking and gas-exchange analysis, the germinated seeds were transferred to a pot containing Levington F2S seed & modular compost and vermiculite medium (v:v = 3:1). Both WT and transgenic plants were cultured in an environment-controlled chamber (Sanyo, Japan) in air supplemented with 1% (v:v) CO_2_, 25/20 °C day/night, 12/12 h light/dark, and ~150 μmol photons m^−2^ s^−1^ (white light). Net photosynthesis rate (Pn, μmol m^−2^ s^−1^) at 25 °C over the range of internal CO_2_ partial pressure (Ci; μbar) were examined on the same leaves during gas-exchange experiments using the portable flow-through LI-6800 gas-exchange system (LI-COR, Nebraska, USA).

### Protein isolation and characterization

For total protein analysis and measurement of the absolute content of Rubisco, 2-cm^2^ leaves were taken and thoroughly homogenized by pestle and mortar in 1 mL pre-cooled extraction buffer (50 mM 3-[4-(2-Hydroxyethyl)−1-piperazinyl] propanesulfonic acid (EPPS), 20 mM NaHCO_3_, 10 mM MgCl_2_, 1% (w/v) PVPP (polyvinylpolypyrrolidone), 5 mM DTT and 1% (v/v) protease inhibitor cocktail (Sigma-Aldrich, USA), pH = 8.0). In total, 300 μL of homogenized samples were centrifuged at 1000×*g* to remove the cell debris and insoluble proteins, and the supernatant was used for Rubisco content measurement and activity assays. Protein quantification was performed by the Bradford method^79^. After protein denaturation with 4× Loading buffer at 95 °C for 10 min, samples were loaded into SDS-PAGE gels for immunoblot analysis according to standard protocols^32,80^. The rabbit polyclonal anti-CbbL (1:10,000 dilution; Agrisera, AS03 037), anti-His (1:10,000 dilution; Invitrogen, 4E3D10H2/E3), anti-CsoS1A/B/C (1:10,000 dilution; Agrisera AS14 2760), CsoS2 (1:1000 dilution; prepared by Genscript, NJ, USA, using the RGTRAVPPKPQSQG peptide), CsoSCA (1:1000 dilution; prepared by Genscript, NJ, USA, using the RHGGRYPPNDIGHA peptide), the horseradish peroxidase-conjugated goat anti-mouse (1:10,000 dilution; Promega, W4021), and a goat anti-rabbit (1:10,000 dilution; Agrisera AS101461) secondary antibodies were used for immunoblot analysis.

For the purification of CsoSCA, the coding sequence of the *csoSCA* gene was cloned into pBAD33^31^. The plasmid was transformed into *E. coli* BL21(DE3) and single clone was cultured overnight at 37 °C in 5 mL lysogeny broth (LB) medium containing 10 μg mL^−1^ chloramphenicol. The culture was diluted to 200 mL, and arabinose was added to a final concentration of 1 mM when the optical density (OD) of the culture reaches 0.6 to start protein induction at 25 °C overnight. The cells were harvested by centrifugation at 5000×*g* for 10 min and were washed with 10 mL phosphate-buffered saline (PBS, 137 mM NaCl, 2.7 mM KCl, 10 mM Na_2_HPO_4_, 1.8 mM KH_2_PO_4_, pH 7.4) and were finally resuspended in 10 mL of PBS with 1% Protease Inhibitor Cocktail (Melford, UK). The cells were broken by sonication, and cell debris was removed by centrifugation at 10,000×*g* at 4 °C. The supernatant was filtered with a 0.22-μm filter and CsoSCA was purified by His-trap affinity chromatography on an ÄKTA System using PBS with 1 M imidazole as the elution buffer (GE Healthcare Life Sciences, USA), followed by SDS-PAGE verification (Supplementary Fig. 7).

### Chlorophyll content quantification

In all, 2-cm^2^ leaves were punched and extracted with 2 mL extraction buffer (alcohol, acetone, and water (4.5:4.5:1, v:v:v) in the dark at 4 °C overnight. After the leaves completely turn white, the absorption values of extractions at 645 nm and 663 nm were measured by spectrophotometry using a Nanodrop Ds-11 (DeNovix, USA), and chlorophyll contents were finally calculated based on the equations of Lichtenhaler and Wellburn^81^.

### Carboxysome purification

The native carboxysome was isolated from *H. neapolitanus* using sucrose gradient ultracentrifugation^39^, and synthetic carboxysome purification procedures were performed from the transgenic leaves and *E. coli*^31,38,39^. Cell growth was maintained in the Vishniac and Santer growth medium^82^ using a 5-L fermenter (BioFlo 115, New Brunswick Scientific, USA) at 30 °C. The pH of the growth medium was monitored using a pH probe and was maintained at pH 7.6 by constant supplement of 3 M KOH. Air supply was set at 500 L min^−1^ for initial growth and reduced to 200 L min^−1^ 24 h before harvesting. Cells from the 5-L culture were collected by centrifugation and then resuspended in 15 mL of TEMB buffer (5 mM Tris-HCl, 1 mM EDTA, 10 mM MgCl_2_, 20 mM NaHCO_3,_ pH 8.0). After treating with 0.5 mg mL^−1^ chicken egg white lysozyme at 30 °C for 1 h, cells were broken by glass beads (150 to 212 mm, acid washed; Sigma-Aldrich, USA) for 10 min. The cell extracts were further treated with 33% (v/v) B-PERII (ThermoFisher Scientific, USA) and 0.5% (v/v) IGEPAL CA-630 (Sigma-Aldrich, USA) for 2 h. The cell debris was removed by centrifugation at 9000×*g* for 10 min.

Nearly 10 g transgenic leaves were homogenized in 100 mL TEMB containing 1% (v/v) protease inhibitor cocktail (Melford, UK) on ice by a blender. IGEPAL CA-630 was added to a final concentration of 1% (v/v) mixed on a rotary for 60 min at 4 °C to remove the membrane. Leaves extracts were then filtered through a single layer of Miracloth (Merck-Millipore, Bayswater, VIC, Australia) and then centrifuged at 3000×*g* for 1 min to remove starch.

*E. coli* (TOP10) was picked and cultured in 20 ml of LB medium with 10 μg mL^−1^ chloramphenicol at 37 °C overnight. The cultures were diluted in 800 mL of medium in a 2-L flask and arabinose was added to a final concentration of 1 mM to start the protein induction at 25 °C and 120 rpm overnight when the optical density of the culture reached 0.6. Cells were collected at 5000×*g* for 10 min and then washed with 10 mL of TEMB buffer. The cells were resuspended in 20 mL of TEMB buffer containing 10% (v/v) CelLytic B-cell lysis reagent (Sigma-Aldrich, USA) and 1% (v/v) Protease Inhibitor Cocktail (Melford, UK). The cells were broken by sonication and the cell debris was removed by centrifugation at 10,000×*g* for 10 min.

All the crude carboxysome containing supernatants from *H. neapolitanus*, transgenic leaves and *E. coli* cells were centrifuged at 47,000×*g* for 30 min at 4 °C to enrich carboxysomes. The pelleted carboxysomes were resuspended in 2 mL of TEMB and centrifuged at 10,000×*g* for 1 min before loading the supernatant onto a 10, 20, 30, 40, and 50% (w/v) sucrose density step gradient. Different sucrose fractions were collected for further analysis after centrifugation at 80,000×*g* for 30 min at 4 °C.

### Rubisco and carbonic anhydrase activity assays

Rubisco carboxylation activities were conducted according to refs. ^31,39,80,83^. In detail, NaH^14^CO_3_ was added to N_2_-gas bubbled Rubisco activity assay buffer (100 mM EPPS, 20 mM MgCl_2_, (+/-) 50 U mL^−1^ carbonic anhydrase (Sigma-Aldrich, USA; C3934), pH 8.0) to prepare reaction buffer containing 1.5 mM to 50 mM NaH^14^CO_3_ (corresponding to 20–640 μM CO_2_). The concentration of carboxysomes was adjusted to 200 ng µL^−1^ and 5 µL of purified synthetic carboxysomes (1 μg) or total soluble proteins from leaf homogenates were pre-incubated in the reaction buffer for 5 min and the reaction was started by addition of RuBP to 1 mM at 25 °C and terminated after 5 min incubation by adding 10% (v/v) formic acid. Samples were then dried on heat blocks at 100 °C to remove the free NaH^14^CO_3_. The pellets were resuspended with 200 µL distilled water and then mixed with 2 mL scintillation cocktail (Ultima Gold XR, Perkin-Elmer, USA). Radioactivity measurements were carried out using a scintillation counter (Tri-Carb, Perkin-Elmer, USA). Raw readings were converted to the amount of fixed ^14^C according to the standard curve. The measurement was carried out using three independent biological replicates, and the data were fitted with a Michaelis−Menten kinetic model using OriginPro 2020b (OriginLab, USA).

The number of Rubisco active sites was evaluated by inhibition of activity using different amounts of CABP^48^. In brief, 5 µL protein samples were pre-incubated with reaction buffer containing 0, 10, 20, and 40 nM CABP for 15 min at 25 °C, and the carboxylation reaction was then initiated by adding RuBP to 1 mM, and the assay used to determine Rubisco activity was followed. The fitted equations from Rubisco activities as a function of the CABP concentration were used to evaluate the absolute number of Rubisco active sites (Supplementary Fig. 1).

Carbonic anhydrase assays were performed in vitro, based on a potentiometric method^57–60^, to measure the change in the buffer pH as a function of time after the injection of a standard amount of CO_2_-saturated water. Samples were added into 2 mL pre-cooled (0−2 °C) assay buffer (20 mM Tris, pH 8.0), and the reactions were initiated with the supplement of 0.5 mL of ice-cold CO_2_ saturated water (prepared by bubbling with CO_2_ for 30 min). The reaction buffer was continuously stirred, and the change in the buffer pH was measured as a function of time by using a pH microelectrode (Mettler Toledo, 30244732, Switzerland). The catalytic capacities of CA for the CO_2_ hydration reactions were analyzed for intact CB^Tob^, ruptured CB^Tob^ by a freeze-thaw treatment to release encapsulated CA^59^, as well as the control samples in the absence of proteins or containing WT tobacco leaf extract obtained following the carboxysome purification procedure.

### Mass spectrometry

In total, 50 µg of proteins was diluted to 80 µL with 25 m ammonium bicarbonate and then 1% (w/v) RapiGest detergent was added to a final concentration of 0.05% (w/v), and samples were incubated at 80 °C for 10 min with a continuous shaking at 450 rpm. Cysteine reduction was performed by adding 5 µL of 11.1 mg mL^−1^ dithiothreitol (DTT) solution and incubating at 60 °C for 10 min with a shaking at 450 rpm. Alkylation was performed by adding 5 µL 46.6 mg mL^−1^ iodoacetamide and incubating for 30 min in the dark. Digestion was carried out by adding 5 µL of 200 µg mL^−1^ trypsin (at a 50:1 ratio of sample:trypsin) and incubated at 37 °C overnight with end-over-end mixing. In all, 1 µL of trifluoroacetic acid was added to stop trypsin activity and simultaneously degrade RapiGest, and samples were incubated at 37 °C for 45 min. RapiGest degradation products and any insoluble material were removed by 13,000×*g* centrifugation for 15 min at 4 °C. Samples were finally diluted 1 in 10 for analysis. 3 µL diluted sample was loaded onto the trapping column (Thermo Scientific, PepMap100, C18, 300 μm X 5 mm), using partial loop injection, for 7 min at a flow rate of 4 μL min^−1^ with 0.1% (v/v) formic acid. Samples were resolved on the analytical column (Easy-Spray C18 75 µm × 500 mm 2-µm column) using a gradient of 97% A (0.1% formic acid) 3% B (79.95% acetonitrile, 19.95% water, 0.1% formic acid) to 60% A 40% B over 30 min at a flow rate of 300 nL min^−1^. The data-dependent program used for data acquisition consisted of a 70,000 resolution full-scan MS scan with Automatic Gain Control (AGC) set to 3e^6^ ions with a maximum fill time of 200 ms. The 10 most abundant peaks were selected for MS/MS using a 35,000 resolution scan (AGC set to 1e^4^ ions with a maximum fill time of 100 ms) with an ion-selection window of 2 *m*/*z* and normalized collision energy of 30%.

For relative quantification, Progenesis QI for proteomics v4 (Nonlinear Dynamics, Newcastle, UK) was used to perform label-free quantitation (LFQ). Prominent ion features (>600 per chromatogram) were used as vectors to align each data set to a reference chromatogram. An analysis window of 15–105 min and 350–1500 *m/z* was selected. Log-transformed MS data were normalized by inter-sample abundance ratio, and differences in relative protein abundance were investigated using nonconflicting peptides only. MS/MS spectra were exported in Mascot generic format and searched against the NCBI *Halothiobacillus neapolitanus* database (UP000009102, 2353 proteins) using a locally implemented Mascot server (v.2.2.03; www.matrixscience.com).

### Transmission electron microscopy

The carboxysomes purified from transplastomic chloroplasts were characterized by negative-staining TEM^49,50,64^. The carboxysomes (~4 mg mL^−1^) were stained with 3% uranyl acetate on carbon grids (Agar Scientific, UK; AGS160-3) for electron microscopic imaging. The diameters of the carboxysomes were measured with ImageJ following the reported procedure^31,39,50,64^ and were statistically analyzed using OriginPro 2020b. For thin-sectioning TEM, leaf tissues (2 × 2 mm) were cut and fixed in 3% (v/v) glutaraldehyde with 1% (v/v) paraformaldehyde in 0.1 M sodium cacodylate for thin-sectioning to characterize chloroplast ultrastructures. Leaves were processed by Pelco BioWave Pro laboratory microwave system and fixed by three steps of 100 W for 1 min each. The fixed leaves were then washed three times in 0.1 M sodium cacodylate buffer (pH 6.8), and then a secondary fixation was performed in 0.5% (w/v) osmium tetraoxide in 0.1 M sodium cacodylate. Leaves were then incubated at 100 W for 12 min and serially dehydrated and embedded in LR white resin. Finally, leaf tissue was cut into 70–80 nm ultrathin resin sections and stained with 2% (w/v) uranyl acetate and lead citrate. Leaf sections and purified carboxysomes were imaged at 120 kV using a FEI120kV Tecnai G2 Spirit BioTWIN TEM with a Gatan Rio 16 camera.

### Statistics and reproducibility

For statistics and reproducibility, all the tests in this study were performed at least three times independently.

### Reporting summary

Further information on research design is available in the Nature Portfolio Reporting Summary linked to this article.

## Supplementary information

Supplementary Information

Description of Additional Supplementary Files

Supplementary Data 1

Supplementary Data 2

Reporting Summary

## Data Availability

Data supporting the findings of this work are available within the paper and its Supplementary Information files. A reporting summary for this Article is available as a Supplementary Information file.  Source data are provided with this paper.

## Source data

Source Data
