# Supplementary material for: Engineering α-carboxysomes into plant chloroplasts to support autotrophic photosynthesis
Source: Nat Commun. 2023 Apr 25;14:2118. doi: 10.1038/s41467-023-37490-0 (PMC10130085; doi:10.1038/s41467-023-37490-0)
Supplement: Supplementary file 1 — Supplementary Information [file 41467_2023_37490_MOESM1_ESM.pdf]

Engineering α-carboxysomes into plant chloroplasts to support autotrophic photosynthesis

Chen *et al.*

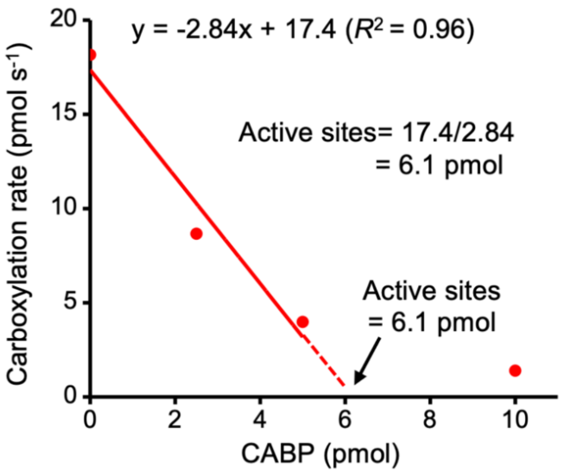

Supplementary Figure 1. Quantification of Rubisco active sites. Absolute Rubisco active sites were evaluated by the Rubisco activities with a function of CABP concentrations (0, 2.5, 5, and 10 pmol), according to the previously reported procedure [1]. The results demonstrated that CABP inhibition is a linear model within a certain concentration range (y = -2.84x + 0.0174, R^2 = 0.96). The X-intercept indicates the concentration of Rubisco active (6.1 pmol). Source data are provided as a Source Data file.

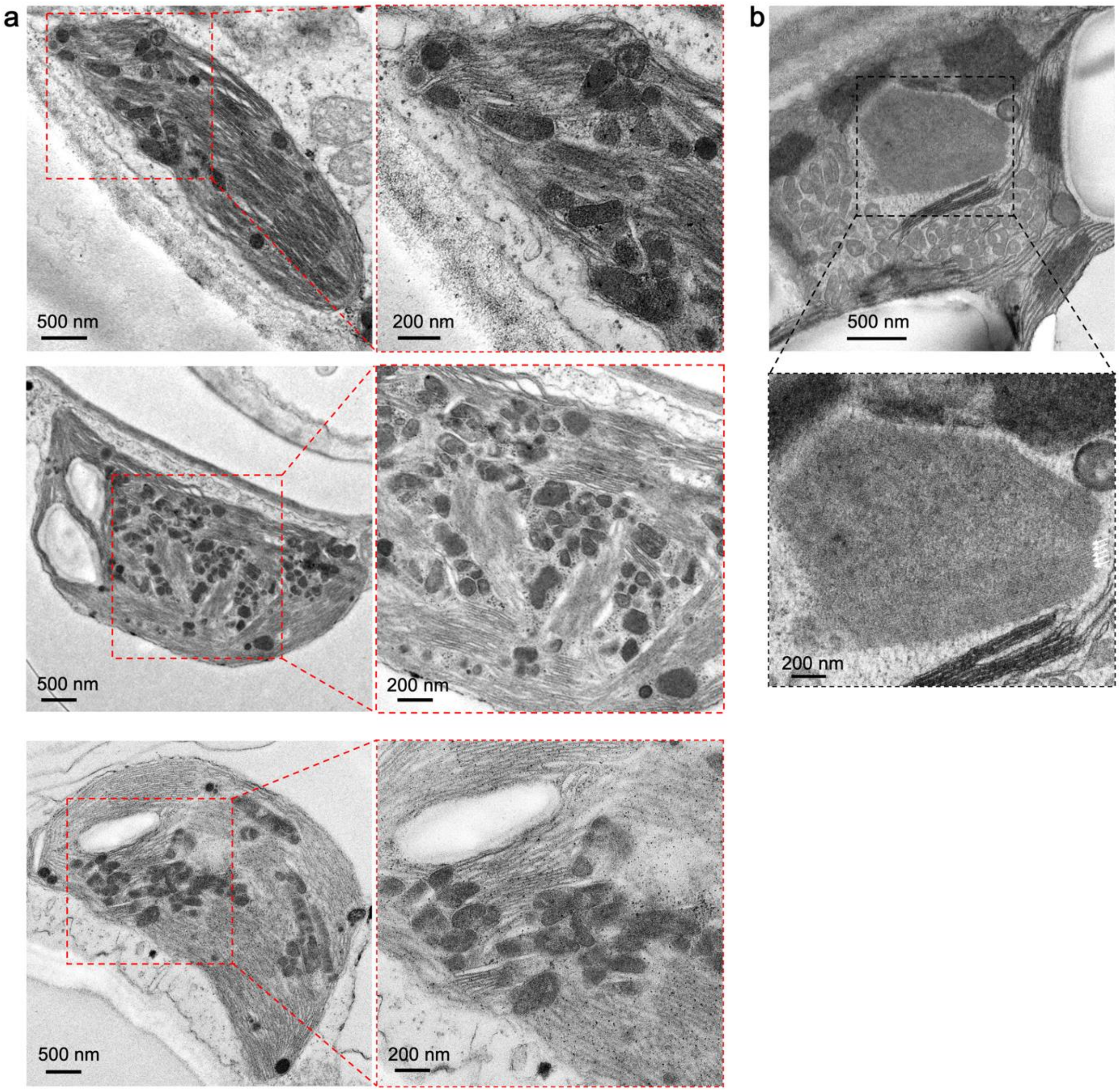

Supplementary Figure 2. Ultra-structures formed by α-carboxysome protein assemblies in tobacco chloroplasts, including canonical α-carboxysome structures and elongated shapes (a), as well as Rubisco assemblies (b). White arrows in (**b**) indicate the paracrystalline arrays of Rubisco in the large assemblies observed in chloroplasts. At least three biologically independent samples were analyzed.

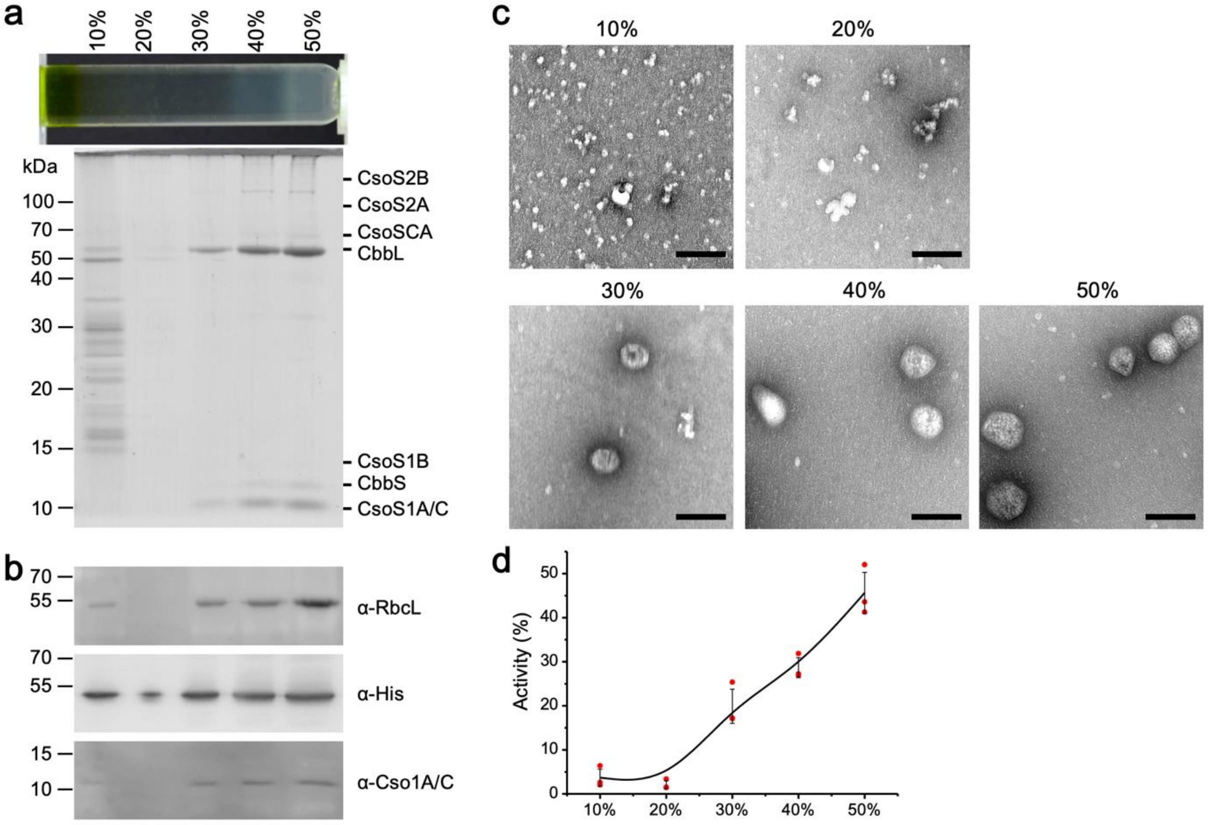

Supplementary Figure 3. Purification and characterization of carboxysomes from the transgenic line. Sucrose gradient ultracentrifugation and SDS-PAGE (**a**), immunoblot analysis (**b**), EM (**c**), and Rubisco activity assays (**d**) of protein samples in different sucrose fractions. **d**, The total activities of 10% to 50% fractions were set as 100%. Data are presented as mean ± SD (n = 3) from three independent biological replicates. These results indicated that functional carboxysomes were enriched in the 50% sucrose fraction. Scale bar = 200 nm in (**c**). Source data are provided as a Source Data file.

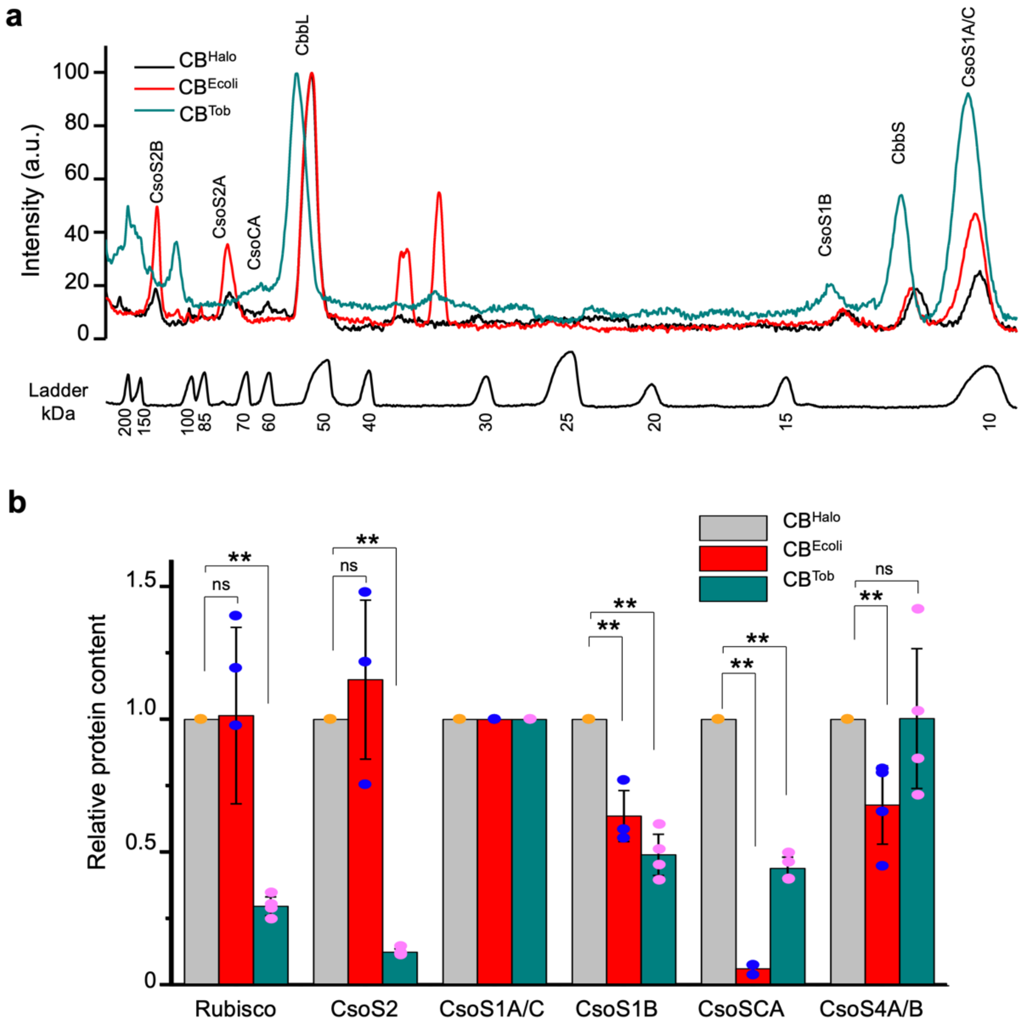

Supplementary Figure 4. The content of individual proteins of carboxysomes. a, SDS-PAGE lane profile analysis shows the differences in the content of individual carboxysome components. The intensities of each Lane were collected from the SDS-PAGE images in Figure 3a and then normalised by the density of CbbL. It is notable that the sizes of proteins are slightly greater in CB^{Tob} compared with those in CB^{Halo} and CB^{Ecoli}, presumably due to post-translation modifications occurred in higher plants [2] (Supplementary File 2). **b**, The relative protein content of CB^{Tob} quantified by mass spectrometry (See also Supplementary File 3) in comparison with those of CB^{Halo} and CB^{Ecoli} as previously reported [3], normalized by the abundant shell hexamers CsoS1A/C (which could be detected by the anti-CsoS1A/C antibody in immunoblot analysis). The protein content of shell pentamers CsoS4A/B in CB^{Tob} and CB^{Halo} was consistent. The abundance of the core components (Rubisco, CsoS2 and CsoSCA) and CsoS1B was reduced in CB^{Tob} than in CB^{Halo}. The CsoSCA content in CB^{Tob} is ~45% of that in native CB^{Halo} and 5-fold than CB^{Ecoli}. Data are presented as mean ± SD (n = 4) from four independent biological replicates and normalised against the content of CsoS1A/C shell proteins (the p values of Rubisco, CsoS2, CsoS1B, CsoSCA and CsoS4A/B are 0.9441/3.9642e^{-8}, 0.2236/2.1668e^{-11}, 0.0014/2.7748e^{-5}, 3.0003e^{-9}/ 4.4770e^{-7} and 0.0091/0.9849 in (CB^{Halo}/CB^{Ecoli})/(CB^{Halo}/CB^{Tob}), respectively). *ns*, $p > 0.05$; *, $p < 0.05$; **, $p < 0.01$ (two-sided t-test). Source data are provided as a Source Data file.

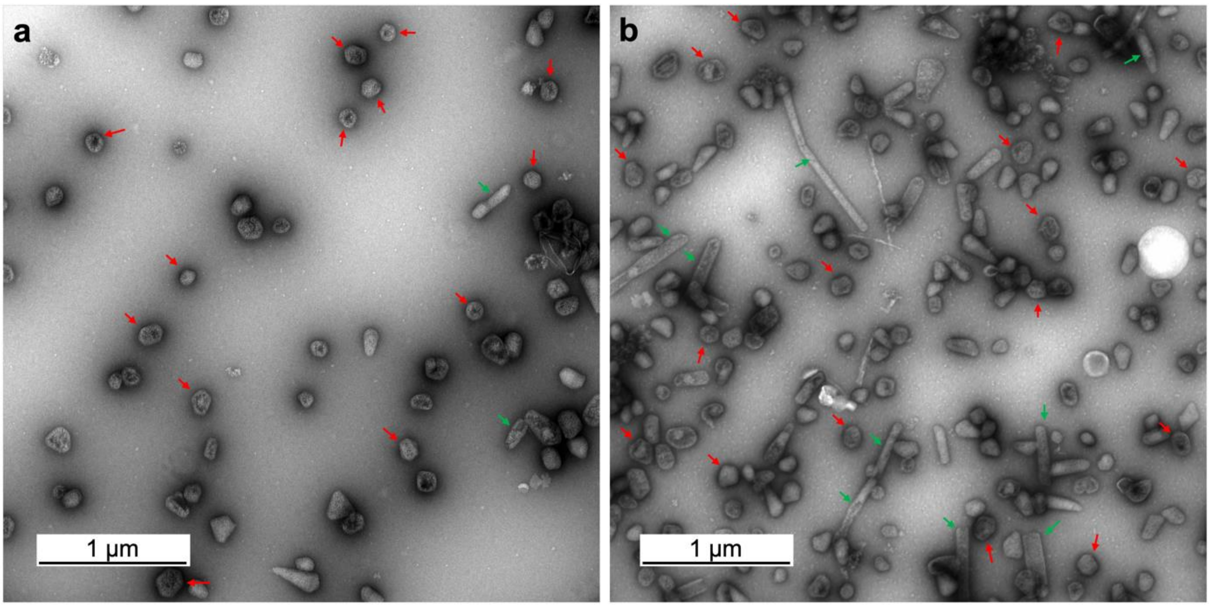

Supplementary Figure 5. EM images of carboxysome polyhedral structures (red arrows) along with elongated structures (green arrows) in the 50% sucrose fraction. **a**, Raw EM image of Figure 3c (CB^{Tob}). **b**, Elongated structures were observed along with polyhedral structures. At least three biologically independent samples were analyzed.

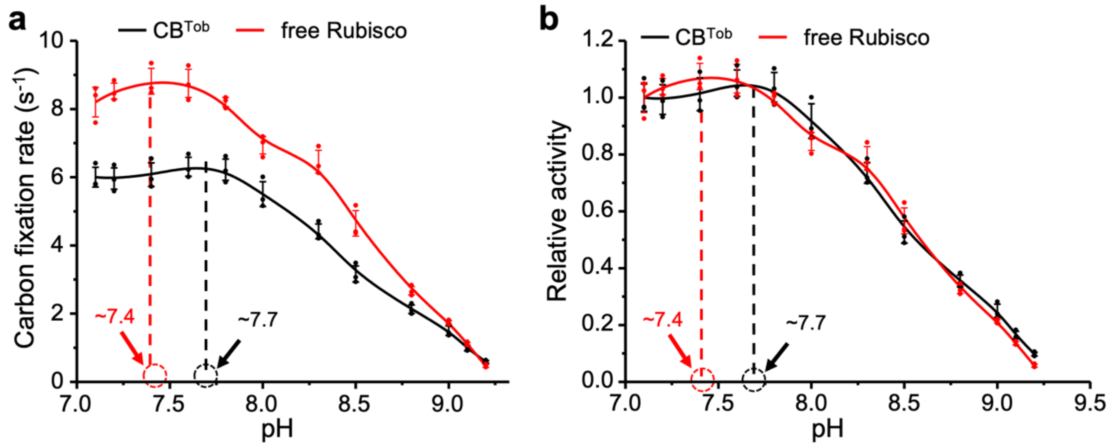

Supplementary Figure 6. Changes in the CO_2-fixing activities at different pH. a. Carbon-fixation activities of CB^{Tob} and free Rubisco in the pH range of 7.1-9.2. The CO_2-fixing activity of CB^{Tob} slightly increased from pH 7.1 to pH 7.4 (for free Rubisco) or pH 7.7 (for CB^{Tob}) and then decreased with the increase in pH to pH 9.2. The observed decreases in the CO_2-fixing activities of CB^{Tob} and free Rubisco at higher pH were reminiscent of the performance of spinach Rubisco [4]. The assays were performed with 50 mM $NaH^{14}CO_3$ and 1 mM RuBP at 25°C. Data are presented as mean ± SD (*n* = 3) from three independent biological replicates. All data were normalized against the number of Rubisco active sites, which was determined by the activity inhibition by gradient amounts of CABP as described in Supplementary Figure 1 and Materials and Methods. **b**. Relative CO_2-fixing activities normalized by the measured activities at pH 7.1. A notable peak shift was observed (also indicated in panel **a**), suggesting that the CB^{Tob} shell could generate a more acidic pH microenvironment within CB^{Tob} than external pH consistent with recent studies [5], and thereby, the functional integrity of the CB^{Tob} shell. Data are presented as mean ± SD (*n* = 3) from three independent biological replicates. Source data are provided as a Source Data file.

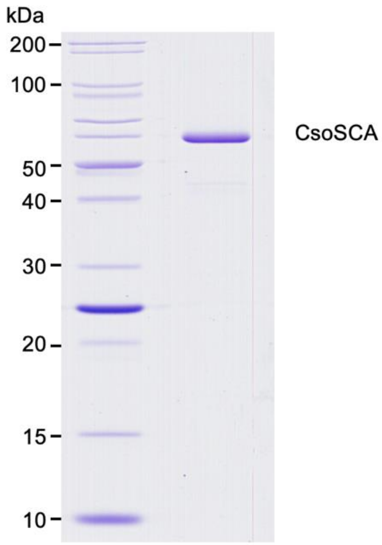

Supplementary Figure 7. SDS-PAGE of purified CsoSCA. At least three biologically independent samples were analyzed.

Supplementary Table 1. The relative protein content of chloroplast-expressed carboxysomes in four biological replicates quantified by label-free mass spectrometry.

	$CB^{Halo\ 1}$				$CB^{Ecoli\ 6}$				CB^{Tob}			
	**R1**	**R2**	**R3**	**R4**	**R1**	**R2**	**R3**	**R4**	**R1**	**R2**	**R3**	**R4**
CbbL	2.87E+09	2.21E+09	2.03E+09	2.28E+09	1.78E+09	1.82E+09	1.47E+09	4.33E+08	4.25E+08	3.05E+08	3.37E+08	3.30E+08
CbbS	4.88E+08	6.30E+08	6.08E+08	5.76E+08	3.54E+08	4.41E+08	3.08E+08	1.05E+08	2.84E+08	2.10E+08	1.85E+08	1.75E+08
CsoS2	4.38E+08	4.62E+08	4.27E+08	4.21E+08	4.21E+08	4.58E+08	3.57E+08	1.28E+08	3.75E+07	3.12E+07	3.53E+07	3.62E+07
CsoS1A/C	5.10E+08	4.96E+08	5.68E+08	4.25E+08	6.50E+08	4.05E+08	3.22E+08	5.78E+07	3.74E+08	2.97E+08	3.24E+08	3.04E+08
CsoS1B	4.04E+07	5.43E+07	5.46E+07	5.43E+07	2.85E+07	2.59E+07	2.38E+07	1.15E+07	1.79E+07	1.47E+07	1.59E+07	1.53E+07
CsoSCA	4.34E+07	3.54E+07	3.76E+07	3.59E+07	2.06E+06	2.13E+06	1.57E+06	3.53E+06	1.27E+07	9.78E+06	1.06E+07	1.02E+07
CsoS4A	7.04E+05	6.49E+05	5.07E+05	9.54E+05	5.09E+05	3.53E+05	3.11E+05	9.89E+04	1.48E+05	1.93E+05	1.98E+05	2.24E+05
CsoS4B	2.99E+05	2.61E+05	2.40E+05	2.51E+05	6.25E+04	1.31E+05	2.57E+04	3.44E+04	4.78E+05	3.69E+05	4.04E+05	3.91E+05
CsoS4A/B	1.00E+06	9.11E+05	7.47E+05	1.21E+06	5.71E+05	4.84E+05	3.37E+05	1.33E+05	6.25E+05	5.62E+05	6.02E+05	6.15E+05

Data represents average normalized abundances of individual components in individual biological replicates.

Supplementary Table 2. Primers used in this study.

Name	Sequence (5' to 3')
pBAD33-F	GCCAGGATCCGAATTCGAGCT
pBAD33-R	CATGGTATATCTCCTTCTTGAATTCGCTAGC
CsoSCA-F	TAGCGAATTCAAGAAGGAGATATACCATGAACACCCGTAACACACGAAGC
CsoSCA-R	AGCTCGAATTCGGATCCTGGCTCAGTGGTGATGATGGTGATGTGCGGATGCAACCTCTTCAATC
operon1-F	GAGTTGTAGGGAGGGATTTGTCGAATGGCAGTTAAAAAGTATAGTGC
operon1-R	CCCCTTCCCCTTACCTAAATATATAGAATAGAGGAATCCCGATTTGTCAAGTCTC
operon2-F	CTATATATTTAGGTAAGGGGAAGGGGAAAACAATTATTATTTTACTGCG
operon2-R	CTTTTCTTGGATCCAATTCAATGGAACAATGATAAAAAAATACAAATAGAAAAGG
operon3-F	GTTCCATTGAATTGGATCCAAGAAAAGTGAGCTATTAACGCG
operon3-R	CCCAATTCGGGATCTGTCGATTAATTACATACCATCCATACATAG

Supplementary references

1. Davidi D*, et al.* Highly active rubiscos discovered by systematic interrogation of natural sequence diversity. *EMBO J*, **39**, e104081 (2020).
2. Mann M, Jensen ON. Proteomic analysis of post-translational modifications. *Nat Biotechnol* **21**, 255-261 (2003).
3. Sun Y*, et al.* Decoding the absolute stoichiometric composition and structural plasticity of α-carboxysomes. *mBio* **13**, e0362921 (2022).
4. Mott KA, Berry JA. Effects of pH on activity and activation of ribulose 1,5-bisphosphate carboxylase at air level CO_2. *Plant Physiol* **82**, 77-82 (1986).
5. Huang J*, et al.* Probing the internal pH and permeability of a carboxysome shell. *Biomacromolecules* **23**, 4339-4348 (2022).
6. Sun Y*, et al.* Decoding the absolute stoichiometric composition and structural plasticity of α-carboxysomes. *mBio* **13**, e0362921 (2022).
