# Supplementary material for: Engineering α-carboxysomes into plant chloroplasts to support autotrophic photosynthesis
Source: Nat Commun. 2023 Apr 25;14:2118. doi: 10.1038/s41467-023-37490-0 (PMC10130085; doi:10.1038/s41467-023-37490-0)
Supplement: Supplementary file 2 — Description of Additional Supplementary Files [file 41467_2023_37490_MOESM2_ESM.pdf]

Description of Additional Supplementary Files

File Name: Supplementary Data 1
Description: Nucleotide sequences of synthesized operons for the expression of α-carboxysome proteins in tobacco chloroplasts.

File Name: Supplementary Data 2
Description: Mass spectrometry data of identified unique peptides of purified carboxysomes (CB^{Tob}) generated from tobacco chloroplasts.
