# Supplementary material for: Engineering α-carboxysomes into plant chloroplasts to support autotrophic photosynthesis
Source: Nat Commun. 2023 Apr 25;14:2118. doi: 10.1038/s41467-023-37490-0 (PMC10130085; doi:10.1038/s41467-023-37490-0)
Supplement: Supplementary file 5 — Reporting Summary [file 41467_2023_37490_MOESM5_ESM.pdf]

nature portfolio

Corresponding author(s): Professor Luning Liu

Last updated by author(s): Mar 5, 2023

Reporting Summary

Nature Portfolio wishes to improve the reproducibility of the work that we publish. This form provides structure for consistency and transparency in reporting. For further information on Nature Portfolio policies, see our Editorial Policies and the Editorial Policy Checklist.

Statistics

For all statistical analyses, confirm that the following items are present in the figure legend, table legend, main text, or Methods section.

n/a	Confirmed	
☐	☒	The exact sample size (*n*) for each experimental group/condition, given as a discrete number and unit of measurement
☐	☒	A statement on whether measurements were taken from distinct samples or whether the same sample was measured repeatedly
☐	☒	The statistical test(s) used AND whether they are one- or two-sided<br>*Only common tests should be described solely by name; describe more complex techniques in the Methods section.*
☒	☐	A description of all covariates tested
☒	☐	A description of any assumptions or corrections, such as tests of normality and adjustment for multiple comparisons
☐	☒	A full description of the statistical parameters including central tendency (e.g. means) or other basic estimates (e.g. regression coefficient) AND variation (e.g. standard deviation) or associated estimates of uncertainty (e.g. confidence intervals)
☐	☒	For null hypothesis testing, the test statistic (e.g. *F*, *t*, *r*) with confidence intervals, effect sizes, degrees of freedom and *P* value noted<br>*Give P values as exact values whenever suitable.*
☒	☐	For Bayesian analysis, information on the choice of priors and Markov chain Monte Carlo settings
☒	☐	For hierarchical and complex designs, identification of the appropriate level for tests and full reporting of outcomes
☒	☐	Estimates of effect sizes (e.g. Cohen's *d*, Pearson's *r*), indicating how they were calculated

Our web collection on statistics for biologists contains articles on many of the points above.

Software and code

Policy information about availability of computer code

Data collection	Transmission electron microscope (TEM) were imaged by FEI120kV Tecnai G2 Spirit BioTWIN TEM with a Gatan Rio 16 camera. Plant gas exchange analysis were carried out by LI-6800 (LI-COR, United States).
Data analysis	The size of carboxysomes were measured by using image J (1.51n). MS/MS spectra were searched by a locally implemented Mascot server (v.2.2.03; www.matrixscience.com).All the figures were generated by OriginPro 2020b (OriginLab, United States) based on the source data.

For manuscripts utilizing custom algorithms or software that are central to the research but not yet described in published literature, software must be made available to editors and reviewers. We strongly encourage code deposition in a community repository (e.g. GitHub). See the Nature Portfolio guidelines for submitting code & software for further information.

Data

Policy information about availability of data

All manuscripts must include a data availability statement. This statement should provide the following information, where applicable:

- Accession codes, unique identifiers, or web links for publicly available datasets
- A description of any restrictions on data availability
- For clinical datasets or third party data, please ensure that the statement adheres to our policy

All data needed to evaluate the conclusions in the paper are present in the main text or the supplementary materials. Source data are provided with the paper. The MS data alignment were performed based on Halothiobacillus neapolitanus database (UP000009102, 2353 proteins).

Human research participants

Policy information about studies involving human research participants and Sex and Gender in Research.

Reporting on sex and gender	N/A
Population characteristics	N/A
Recruitment	N/A
Ethics oversight	N/A

Note that full information on the approval of the study protocol must also be provided in the manuscript.

Field-specific reporting

Please select the one below that is the best fit for your research. If you are not sure, read the appropriate sections before making your selection.

[x] Life sciences [ ] Behavioural & social sciences [ ] Ecological, evolutionary & environmental sciences

For a reference copy of the document with all sections, see nature.com/documents/nr-reporting-summary-flat.pdf

Life sciences study design

All studies must disclose on these points even when the disclosure is negative.

Sample size	As the transgenic Lines were created by homologous recombination based chloroplast transformation, three different transgenic Lines should be sufficient for analysis. Three different plants for both WT and TobCB transgenic lines were cultured to collect the growth data and perform Gas exchange analysis.
Data exclusions	Representative TEM images were presented in the main text and representative carboxysome particles were selected for size measurement. The carboxysomes with various structures were shown in supplementary figures.
Replication	All the measurements were performed in three different biological replicates and were reproduced successfully.
Randomization	Plants were germinated and selected randomly for further growth. As the growth of transgenic Lines were slower than that of WT, transgenic lines and WT were not germinated at the same time. When the heights of WT and transgenic lines were nearly 20 cm, the leaves with similar sizes were collected and chosen randomly for protein measurement and gas exchange analysis.
Blinding	All the experiments were analyzed by the same investigator and the plants were cultured in a controlled conditions. Thus, blinding is not necessary in this research.

Reporting for specific materials, systems and methods

We require information from authors about some types of materials, experimental systems and methods used in many studies. Here, indicate whether each material, system or method listed is relevant to your study. If you are not sure if a list item applies to your research, read the appropriate section before selecting a response.

Materials & experimental systems

n/a	Involved in the study	
[ ]	[x]	Antibodies
[x]	[ ]	Eukaryotic cell lines
[x]	[ ]	Palaeontology and archaeology
[x]	[ ]	Animals and other organisms
[x]	[ ]	Clinical data
[x]	[ ]	Dual use research of concern

Methods

n/a	Involved in the study	
[x]	[ ]	ChIP-seq
[x]	[ ]	Flow cytometry
[x]	[ ]	MRI-based neuroimaging

Antibodies

Antibodies used	Polyclonal anti-CbbL (Agrisera, AS03 037);anti-His (Invitrogen, 4E3D10H2/E3); anti-CsoS1A/B/C (Agrisera AS14 2760); the horseradish peroxidase-conjugated goat anti-mouse (Promega, W4021), goat anti-rabbit (Agrisera AS101461), home-made CsoS2 antibody

(Genscript); home-made CsoSCA antibody (Genscript)

Validation

The antibodies of anti-CbbL (https://www.agrisera.com/en/artiklar/-rbcl-rubisco-large-subunit-form-i-rabbit.html), anti-His (https://www.thermofisher.com/antibody/product/6x-His-Tag-Antibody-clone-4E3D10H2-E3-Monoclonal/MA1-135), and anti-CsoS1A/B/C (https://www.agrisera.com/en/artiklar/csos1a_b_c-major-carboxysome-shell-protein-1a-ab-1c.html) are commercial antibodies and have been widely used in our and other labs. Home-made antibodies were produced by Genscript and have been verified to be able to differentiate target proteins from other proteins in the samples (Figure 3b).
